# Current Advances of Tubulin Inhibitors in Nanoparticle Drug Delivery and Vascular Disruption/Angiogenesis

**DOI:** 10.3390/molecules21111468

**Published:** 2016-11-02

**Authors:** Souvik Banerjee, Dong-Jin Hwang, Wei Li, Duane D. Miller

**Affiliations:** Department of Pharmaceutical Sciences, University of Tennessee Health Science Center, 881 Madison Ave. Memphis, TN 38163, USA; sbanerj5@uthsc.edu (S.B.); dhwang@uthsc.edu (D.-J.H.)

**Keywords:** tubulin dynamics, tubulin inhibitors, multidrug resistance, nanoparticle formulations, β-tubulin isotypes and drug resistance, angiogenesis, vascular disrupting agent, antimitotic

## Abstract

Extensive research over the last decade has resulted in a number of highly potent tubulin polymerization inhibitors acting either as microtubule stabilizing agents (MSAs) or microtubule destabilizing agents (MDAs). These inhibitors have potent cytotoxicity against a broad spectrum of human tumor cell lines. In addition to cytotoxicity, a number of these tubulin inhibitors have exhibited abilities to inhibit formation of new blood vessels as well as disrupt existing blood vessels. Tubulin inhibitors as a vascular disrupting agents (VDAs), mainly from the MDA family, induce rapid tumor vessel occlusion and massive tumor necrosis. Thus, tubulin inhibitors have become increasingly popular in the field of tumor vasculature. However, their pharmaceutical application is halted by a number of limitations including poor solubility and toxicity. Thus, recently, there has been considerable interests in the nanoparticle drug delivery of tubulin inhibitors to circumvent those limitations. This article reviews recent advances in nanoparticle based drug delivery for tubulin inhibitors as well as their tumor vasculature disruption properties.

## 1. Introduction

**Figure 1:**
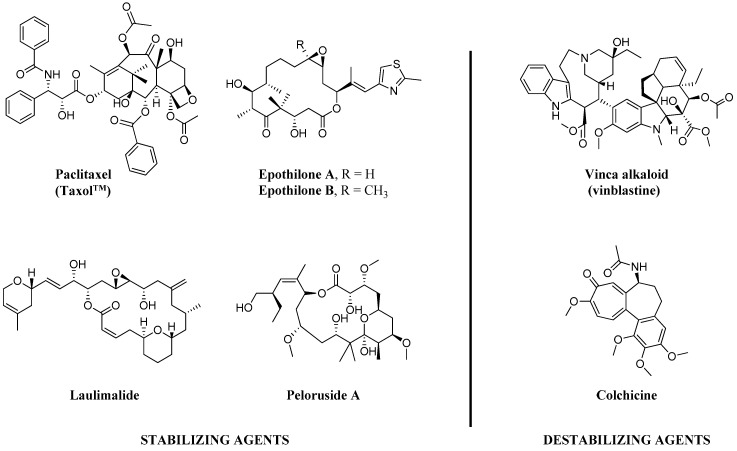
Chemical structures of representative natural products of microtubule stabilizing (MSA) and destabilizing agents (MDA).

Since the discovery of the action of colchicine by Borisy et al. [1] in 1967, for the last 50 years, tubulin/microtubules have been long thought to be crucial chemotherapy targets in various cancer types, especially for breast, lung, ovarian and pancreatic carcinomas [2]. Microtubule-targeted agents (MTAs), including taxanes (e.g., paclitaxel (PTX)) and Vinca alkaloids (e.g., vinblastine) as shown in Figure 1, are considered to work primarily by increasing or decreasing the cellular microtubule mass. These effects play important roles in their chemotherapeutic actions to mitotic block and triggering apoptosis [2]. Additionally, while MTAs are mitotic inhibitors, recent findings suggest that anti-cancer properties of the MTAs may be attributed to their non-mitotic effects [3].

The microtubules are multifunctional cytoskeletal proteins, composed of α- and β-tubulin heterodimers [4], involved in many essential cell functions including maintenance of cell shape, intracellular transport, and in mitosis, functioning as part of the spindle to ensure proper chromosome segregation and cell division [5,6]. Microtubule-targeting agents can be divided into two main separated groups depending on their mechanisms of actions, microtubule-stabilizing (MSA) and microtubule destabilizing agents (MDA) [7]. MSAs prefers to bind to the polymerized tubulins and stabilize microtubules, while MDAs prefer to bind to the tubulin dimers and destabilize microtubules [8,9].

**Figure 2:**
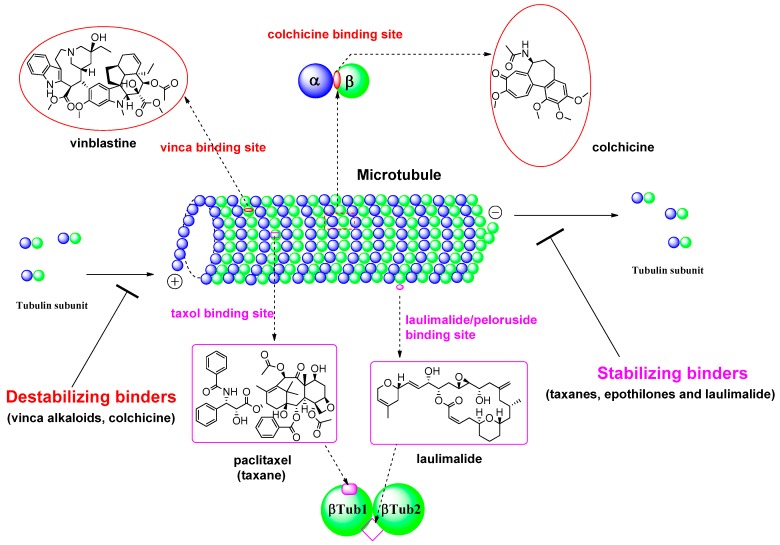
Tubulin binding sites and representative natural products of microtubule-targeted drugs.

During the last 20 years, many MSAs have entered the clinical trial stages and some of them have become effective anticancer drugs [7]. Most of these tubulin inhibitors were derived from natural products or their structural modified analogs. The anti-tubulin/anti-mitotic agents bind to one of the three best characterized binding sites on α- or β-tubulin subunits, which are the taxane, vinca alkaloids, and colchicine binding sites [10]. Recently, Prota et al. [11] reported the anti-tubulin mechanisms of laulimalide and peloruside A by X-ray crystallography. These two MSAs bind to a unique non-taxane site on β-tubulin using their respective macrolide core structures. It showed the agents/ligands interact with a second tubulin dimer across proto-filaments. And they allosterically stabilize the taxane-site M-loop that establishes lateral tubulin contacts in microtubules. The binding modes in each β-tubulin depicted in βTub1 and βTub2 at Figure 2. For MDA, vinca alkaloids, including vinblastine, vincristine, and vinorelbine, promote to protect polymerization of tubulin to destabilize microtubules’ action. The vinca-binding domain on β-tubulin is located near the exchangeable GTP binding site [12]. To treat the patients with the solid tumors or hematologic malignancies, the vinca alkaloids have been used as single agents or in combination with other cytotoxic agents. And, as another destabilizer of MDA, colchicine has been focus on its dynamic instability as small molecules. Ravelli et al. [13] reported a complex of tubulin-colchicin vs. SLD (stathmin-like domain) and its tubulin regulation on crystallization study. It showed the colchicine bound to β-subunits at the interface with α-tubulin. The complex includes two tubulin heterodimers, with colchicine bound to β-subunits at the interface with α-subunit as summarized in Figure 2. For last few decades after a discovery of tubulin action, many microtubule inhibitors of MSAs and/or MDAs have been used for clinical activity to treat aggressive tumors based on their unique mechanisms of action. Some microtubule-targeted drugs can act as vascular-targeting agents [14,15], rapidly depolymerizing microtubules of newly formed vasculature to shut down the blood supply to tumors [16].

Although antitubulin/antimitotic agents are widely used clinically, they have been facing a number of challenges, namely multidrug resistance (MDR) [17], low bioavailability, poor solubility, high toxicity [8], in their clinical trials. To overcome the barrier of current inconvenient for its treatment, a variety of studies have focused on improving the pharmaceutical properties of their agents. As followed our previous review about overview of colchicine binding agents [18], this review provides an overview of its nanoparticle drug delivery and angiogenesis of those microtubule-targeting drugs which are to date under clinical evaluation.

## 2. Nanoparticle Delivery of Tubulin Inhibitors

Recently, a number of research groups both from academia and industry have come up with a number of highly potent tubulin polymerization inhibitors with promising in vitro as well as in vivo efficacy [18,19,20,21,22,23,24,25]. However, the pharmaceutical development of most of the newly developed tubulin inhibitors is facing a number of obstacles, namely: (a) low solubility; (b) low bioavailability, as well as (c) high toxicity [26,27,28,29]. One of the possibilities to circumvent this obstacle is targeted delivery of the tubulin inhibitors to the cancer tissue [29]. There are a number of commonly used measures to targeted drug delivery including polymer conjugation and conjugation to antibodies that are upregulated in specific cancer cells [30,31,32,33,34]. In this context, nanoparticles or macromolecular carries have been observed to introduce positive changes in the pharmacokinetics as well as pharmacodynamics of the chemotherapeutics through a number of ways including: (a) improving solubility; (b) enhancing half-life by avoiding first pass kidney clearance; (c) greatly augmenting drug concentration in tumor cells by improved permeability and retention effect; as well as (d) saving the drug from enzymatic degradation [26,28,29].

### 2.1. Nano Particle Delivery of Tubulin Inhibitors Targeting Vinca Binding Site

#### 2.1.1. Delivery of Tubulysine A (TubA)

Recently, Schluep et al have reported the synthesis and evaluation both in vitro and in vivo of a nanoparticle prodrug of TubA [29]. Tubulysins are one of the naturally available tetrapeptides with highly potent cytotoxic activity against diverse cancer cell lines [29,35,36,37,38,39,40,41]. Tubulysins exert its biological effects by binding to the vinca site of tubulin receptor in a noncompetitive fashion [29,35,36,37,38,39,40]. Recent literature reports a few of articles with total synthesis and structure activity relationship of a number of tubulysin derivatives with equal or greater activity as compared to the naturally occurring tubulysin [38,39,40,41]. However, their pharmaceutical development faces big challenge from their low solubility and high toxicity in animal models [29]. Thus, Schluep et al. had made an effort to covalently attach a thiol derivative of TubA to a linear hexacyclodextrin-based polymer via a disulfide linker leading to stable nanoparticles (CDP-TubA) [29]. Upon achieving the nanoparticles, their tubulin polymerization inhibition as well as anti-proliferative activity were tested in vitro. Additionally, in vivo efficacy of CDP-TubA was evaluated by i.v. administration in the nude mice bearing human HT29 colorectal and H460 non-small cell lung carcinoma tumors. CDP-TubA showed minimum inhibition as compared to the bioactive TubA and the active thiol derivative, indicating prerequisite of the release of peptide drug from the nanoparticle for the targeted inhibition. In vivo evaluation of CDP-TubA using a maximum tolerated dose of 6 mg/Kg showed a potent antitumor effect and significantly prolonged survival compared with TubA alone. These findings suggest that CDP-TubA nanoparticles are highly promising and effective way for the safe delivery of the tubulysin for the treatment of cancer.

#### 2.1.2. Folate Mediated Delivery of Nanoparticle-Loaded Emtansine

Maytansine and its analogues, including emtansine (DM1), have been known to be powerful antimitotic agents capable of depolymerizing microtubules by binding to the tubulin receptor at the vinca binding site [42,43,44]. Maytansine analogues have anti-proliferative activity both in vitro as well as in vivo against a broad spectrum of cancer cell lines including breast cancer, lung carcinoma and murine melanocarcinoma solid tumors [45]. However, therapeutic applications of maytansine and its analogues have been limited by the consequent neurotoxicity and gastrointestinal adverse reactions produced by these analogues [45,46,47]. In recent years, folate has been broadly accepted as a ligand for the targeted delivery of nanoparticle loaded drugs to the folate receptor positive tumors [45,48,49,50]. Folate receptors overexpress in a number of tumor cells including ovary, brain, kidney and breast [45,49,50]. Lately, it has been demonstrated that folate mediated delivery of drug loaded nanoparticles is capable of enhancing binding, improving uptake and increasing cytotoxicity both in vitro as well as in vivo [51,52,53]. In this context, recently, Tang et al. synthesized DM1 loaded PLA-TPGS copolymer nanoparticles immobilized with folates (FA-DM1-NPs) [45]. The FA-DM1-NPs turned out to be highly target specific as demonstrated by the uptake of fluorescent nanoparticles by FR^+^ MCF-7/HER2 cells. The results also exhibit that FA-DM1-NPs induce rapid apoptosis of tumor cells avoiding toxicities and side effects as well as nonspecific distributions exerted by DM1 alone.

#### 2.1.3. α-Cyclodextrin Mediated Delivery of Curcumin to the Cancer Cell

Recently, Jana et al. have demonstrated for the first time that α-cyclodextrin (CD), a polyhydroxy carbohydrate, interacts with the tubulin receptor at the vinblastine binding site utilizing molecular docking and FRET techniques [54]. They have made use of α-cyclodextrin-tubulin interaction to deliver high amount of hydrophobic curcumin to the cancer cell leading to rapid disruption of intracellular microtubules. CD has been known to accommodate diverse poorly water soluble, hydrophobic drugs in its hydrophobic cavity of different sizes from three different kinds of CD (α, β, γ) through formation of inclusion complexes. Thus, CD has been an excellent choice to deliver poorly water soluble drug candidates and currently in use to deliver a number of FDA approved hydrophobic drugs. They have demonstrated that α-cyclodextrin-curcumin complex selectively enters human lung cancer cell (A549) as compared to the human normal lung fibroblast (WI38) and delivers tubulin targeting agent curcumin more close to its binding site at the tubulin surface resulting in apoptotic cell death. These findings point that CD conjugation is highly promising to deliver poorly water soluble hydrophobic tubulin targeting drugs at the tubulin surface.

### 2.2. Nano Particle Delivery of Tubulin Inhibitors Targeting Colchicine Binding Site

#### 2.2.1. Nanoparticle Mediated Delivery of Colchicine Alkaloid

Colchicine was the first known highly potent antimitotic alkaloid and has been widely exploited for the treatment of carcinomas since 1930. Colchicine is known to introduce rapid and severe necrosis and anti-vascular effects in tumors in vivo by depolymerizing microtubule via modulating tubulin receptor [18,55]. Colchicine, thus, has been observed to produce anti-angiogenic effects, given that it should be administered over maximum tolerated dose (MTD) [56,57]. However, it is known to pose fatal toxicity to the patients when administered intravenously [56,58,59]. Thus, recently, there has been considerable research on nanoparticle mediated targeted delivery of colchicine to circumvent the toxicity exerted by it. Recently, Tangutoori et al. [56] reported development and in vitro as well as in vivo characterization of PEGylated Cationic Liposomal-colchicine (PCL-colchicine) nanoparticles for the treatment of lung cancer. One of the primary needs for the PEG coated (PEGylation) nanoparticles is to avoid steric or adhesive hindrances caused by cytoskeletal and cellular organelles in the cytoplasm in order to achieve efficient intracellular transport of nanoparticles [60]. Their results demonstrate that microtubules are more efficiently disrupted by nanoparticle-loaded colchicine. Their in vivo experiments indicate two-fold enhanced accumulation of PCL-colchicine in the malignant lung as compared to the normal lung, yielding longer survival time for the PCL-colchicine treated group.

#### 2.2.2. Delivery of **LY293**

Recently, Mundra et al. reported on the formulation of one of the most potent tubulin inhibitors, the 5-indolyl derivative (2-(1*H*-indol-5-yl) thiazol-4-yl)-3,4,5-trimethoxyphenylmethanone (**LY293**), into a biodegradable co-polymer, mPEG-b-P (CB-co-LA), in an attempt to determine its anticancer activity and mechanism of action [26,28]. **LY293**, discovered by Dr. Miller and Li’s group, has been found to be highly promising for the treatment of resistant melanoma cell line [61,62]. However, the drug is highly hydrophobic, leading to poor solubility [26,28]. The nanoparticles with loaded drugs have been observed to efficiently reduce the proliferation of A375 and B16F10 melanoma cells in vitro through concentration dependent cell cycle arrest in G2/M phase and apoptotic cell death [26,28]. In continuation, the **LY293** loaded nanoparticles showed potent anti-proliferative effect against Pgp overexpressing MDA-MB435/LCC6 MDR1 melanoma cells in vitro and showed the ability to overcome multi drug resistance. Additionally, in vivo experiments with **LY293** loaded nanoparticles exhibited strong inhibition of proliferation of highly aggressive metastasized melanoma mouse model without noticeable toxicity to the important organs. In summary, the **LY293** loaded nanoparticles demonstrated highly promising efficacy against resistance melanoma cells both in vitro and in vivo. These findings strongly advocate for the nanoparticles as an excellent technique for the safe delivery of various highly potent poorly soluble as well as toxic tubulin inhibitors o the specific target.

#### 2.2.3. Delivery of Combretastatin A-4

Combretastatin A-4 (CA4) is an antiangiogenic agent that exert its biological effects by binding to the tubulin receptor at the colchicine binding site. Ten years ago, Sengupta et al. [63] developed PEG-phospholipid copolymer coated PLGA (PEG/PLGA) nanoparticles consisting of a chemo-therapeutic agent doxorubicin (Dox) conjugated to the nanoparticle and an antiangiogenic drug CA4 trapped within the lipid envelope. This staller combination drug delivery system has shown far superior antiproliferative effects than either component of the mixture alone. Their findings suggested that disruption of outer lipid envelope took place inside a tumor leading to rapid deployment of antiangiogenic drug CA4 causing vascular destruction, followed by slow release of the cytotoxic drug Dox from the nanoparticle killing the tumor by increasing apoptotic potential. Recently, Li et al. [64] have come up with a multi-drug delivery system (DDS) based on mesoporous silica nanoparticles (MSNs) exploiting the strategy discovered by Sengupta et al. Li et al. Have demonstrated co-loading of antiangiogenic CA4 and chemotherapeutic Dox in the MSNs followed by anchoring the MSNs onto the iRGD peptide that has been reported to be the ligand with high affinity for α_2_β_3_ receptor overexpressed by a number of tumor cells as well as endothelial cells. Results demonstrated that the antiangiogenic CA4 was released from the DDS rapidly and target specifically at the tumor vasculature as soon as the DDS arrives at the tumor vasculature as guided by the iRGD. Later, upon uptake of DDS by the tumor cells, the chemotherapeutic Dox gets released predominantly within the cells of low pH. This mechanism of action led to significantly improved antiangiogenic and anticancer effects in vivo including complete suppression of tumor growth in three weeks at very low Dox dose. Recently, Sanyal et al. [65] reported development of dendron-polymer conjugates (DPDs) capable of conjugating with anti-angiogenic CA4. They have demonstrated that drug release from the DPDs increases as pH decreases indicating potential improvement in drug release upon internalization in the acidic tumor cells which are low in pH. The DPDs-CA4 construct has shown far superior cytotoxicity than the DPSs alone in the in vitro cellular internalization and toxicity studies. Also, effective antiangiogenic effect of the DPDs-CA4 construct was confirmed by the in vitro endothelial cell tube formation assay.

#### 2.2.4. Delivery of Etoposide

Recently, Athawale et al. reported that the solid lipid nanoparticles (SLN) mediated delivery of etoposide, potent tubulin polymerization inhibitor, circumvented the issues associated with its low solubility as well as the low bioavailability [27]. They have evaluated the etoposide loaded SLN for the in vitro dissolution and cytotoxicity assay as well as for in vivo distribution of the dug. In vivo chemotherapeutic activity of the nanoparticle loaded drug was studied by conducting anti-metastatic activity on a B16F10 melanoma mouse model. The results of the MTT assay demonstrated the prepared nanoparticles showed potent in vitro cytotoxicity in concentration and time dependent manner. The in vitro cytotoxicity results were in concert with the in vitro dissolution assay. This article reports significant improvement in the pharmacokinetic parameters of etoposide upon administration as etoposide-SLN (Et-SLN) nanoparticles. In continuation, the bio distribution pattern exhibited higher distribution of the drug in liver and lung as compared to the etoposide alone. The in vivo anti-metastatic study portrayed significant reduction in the metastasized tumor colonies with the administration of the Et-SLN as compared to the etoposide alone. These findings strongly suggest that Et-SLN technology can be further developed for the treatment of highly metastasizing melanoma without having to worry about the low solidity of etoposide.

### 2.3. Nano Particle Delivery of Tubulin Inhibitors Targeting Paclitaxel Binding Site

#### Delivery of Paclitaxel

Recently, Houghton et al. reported on the successful evaluation of albumin-bound paclitaxel (nab-pac) against Pediatric Preclinical Testing Program (PPTP) solid tumors [66]. Paclitaxel is an antimitotic natural product that stabilizes tubulin by preventing depolymerization and, thus, promoting disturbance of mitotic cellular functions resulting in cell death [67,68,69]. Paclitaxel has broad spectrum antiproliferative activity, including ovarian and breast cancer [66]. However, clinical use of paclitaxel is limited by the toxic effect of anti-mitotic agents on the proliferating cells and poor solubility that comes from the hydrophobicity of paclitaxel [66]. Thus, the original formulation of paclitaxel uses polyoxyethy-lated castor oil (Cremophor EL) known to create severe or fatal hypersensitivity reactions [70,71,72,73,74]. Additionally, cremophor is responsible for peripheral neuropathy and limited penetration of paclitaxel [75,76,77]. The nanoparticle albumin binding drug delivery technology exploits the biochemical properties of albumin to enhance drug delivery to the tumors via albumin introduced transcytosis. The nab-pac, solvent free particle form of paclitaxel, is the first commercial product of this technology [66]. Recently, a Phase 3 clinical trial, that compared the activity and tolerability of nab-pac to that paclitaxel in women with metastatic breast cancer, showed that nab-pac is significantly favored over paclitaxel alone [66,78]. In addition, nab-pac has recently been approved for the treatment of metastatic non-small-cell lung carcinoma in 2012 as well as for metastatic pancreatic cancer in 2013 with significant advantage over solvent based PTX formulation [79,80,81]. Moreover, no severe hypersensitivity reactions occurred in the nab-paclitaxel group, despite the shorter administration time and the absence of premedication. Houghton et al. proved that the activity of nab-paclitaxel against pediatric models of rhabdomyosarcoma and neuroblastoma revealed noticeable in vivo activity superior to that of paclitaxel [66]. In continuation, due to mild and manageable side effects, nab-pac is preferred over PTX in combination with other cytotoxic and targeted agents. Recently, Bansal et al. [82] reported construction of bovine serum albumin (BSA) based nanoparticles mediated delivery of paclitaxel with the ability to controlled release of drug until 24 h. They have also prepared polysorbate 80/Tween 80 (P80) coated BSA-paclitaxel and its blood brain barrier crossing ability was tested in vivo along with the uncoated counterpart as well as the paclitaxel itself. In vivo experiments showed that P80 coated BSA-paclitaxel reached the mouse brain in significantly high concentrations than either the uncoated BSA-paclitaxel or paclitaxel itself. These findings strongly advocate for the nanoparticle ability to cross the blood brain barrier. In the context of brain tumors, lately, Wang et al. [83] have reported development and testing of Pep-1-conjugated PEGylated nanoparticles loaded with paclitaxel (Pep-NP-PTX) as a targeted drug delivery system for the treatment of glioma. They have shown that Pep-NP-PTX system has been uptaken by the glioma cells at significantly higher amount than the NP-PTX system. In vitro cytotoxicity assay has demonstrated two fold higher cytotoxicity of Pep-NP-PTX than NP-PTX system. In continuation, in vivo experiments have suggested that Pep-NP-PTX system has higher distribution in glioma tissues as compared to normal tissues upon administration. Finally, they have found that Pep-NP-PTX system has far superior glioma efficacy resulting in considerable higher survival time (32 days) than either the NP-PTX system (23 days) or he PTX alone (22 days). Recently, Fu and Gong et al. [84] have reported Human Serum Albumin (HAS)-based nanoparticle delivery system for the co-delivery of pirarubicin (THP) and paclitaxel (PTX) for the treatment of breast cancer aiming at reduced toxicities and enhanced therapeutic efficacies. The prepared co-delivery system has demonstrated high loading capacity and sustained release ability. In vivo experiments with 4T1 murine breast cancer cell lines have exhibited significantly higher drug concentrations in tumor cells and lower drug distribution in normal tissues. In vitro cytotoxicity assay as well as in vivo antitumor assays have exhibited far superior antitumor effect of the THP and PTX loaded co-delivery system than either single drug or free combination. Additionally, PTX and THP loaded co-delivery system have demonstrated the ability to induce rapid apoptosis in 4T1 breast cancer cell lines as well as considerably lower side effects. Table 1 summarizes the recent advances in nanoparticle mediated delivery of tubulin inhibitors and the advantages of nanoparticle-drug conjugates as compared to the free drug alone as discussed above.

## 3. Vascular Disrupting Agents and Antiangiogenic Agents

In recent years there has been considerable interest in vascularization for the metastasis and growth of malignant tumors [19,55]. It is scientifically accepted that a solid tumor cannot grow without the blood vessels providing access to the nutrients and oxygen necessary for the tumor survival [55]. Currently, there are two different ways to target tumor vascularization, namely: (a) inhibition of angiogenesis or formation of new blood vessels; and (b) disruption of already existing blood vessels [55,85,86,87]. In recent years, angiogenesis has been extensively studied revealing the cause underlying this process under physiological and pathological conditions [88]. The recent angiogenesis research has provided access to a number of FDA approved drugs, including small molecules and monoclonal antibodies, inhibiting angiogenesis [89]. However, antiangiogenic drugs could not keep up with the expectations as clinical data revealed that treatment with antiangiogenic agents suffer from resistance development, lack of efficacy, as well as toxicity [90]. The second approach, disruption of already existing blood vessels, counts on Vascular Disrupting Agents (VDAs). VDAs are known to promote rapid collapse of tumor vasculature resulting in necrosis at the tumor [19]. The research has proven that antiangiogenic compounds are especially useful at early stage tumors, small tumors, requiring prolong and chronical administration [55]. On the contrary, VDAs are highly cytotoxic and employs its rapid effect on the existing tumor vessels resulting in quick vascular collapse and ultimate tumor cell death [15]. Thus, VDAs are useful against large tumors and require acute administration [15]. Additionally, the VDAs may be more effective for the aggressive tumors that are resistant to chemotherapy and radiotherapy, given VDAs target tumor vasculature. In this review we will be discussing the recent development of VDAs as well as antiangiogenic agents that are tubulin destabilizing agents capable of inducing vascular collapse through rapid depolymerization of microtubules. In recent years, there has been extensive research on the design and development of VDAs as well as antiangiogenic agents that act at the colchicine binding cite [19,23,91,92]. Here, we will primarily be focusing on recent advances on two different classes, namely (1) combretastatin A-4 (CA-4) and its derivatives and (2) non-CA-4 analogues. It is noteworthy that both of the classes belong to the MDA family. There are a number of reports on the antiangiogenic properties of MSAs including paclitaxel, peloruside A as well as laulimalide [93,94,95,96,97,98]. However, antiangiogenic applications of MSAs has faced a number of limitations including (a) resistance to MSAs and (b) neurological as well as bone marrow toxicity [8,94]. In MSA family, paclitaxel (MSA) has been extensively studied, in recent years, for its promising antiangiogenic activity both as a free drug as well as loaded in the nanoparticles [96,98]. However, VDAs and antiangiogenic agents from MDA family had become increasingly popular in recent years in both academic and industrial arena. Thus, here, we will be focusing on the two different classes of VDAs and antiangiogenic agents that belong to the MDA family of tubulin inhibitors.

### 3.1. VDAs and Antiangiogenic Agents from the CA-4 Family

**Figure 3:**
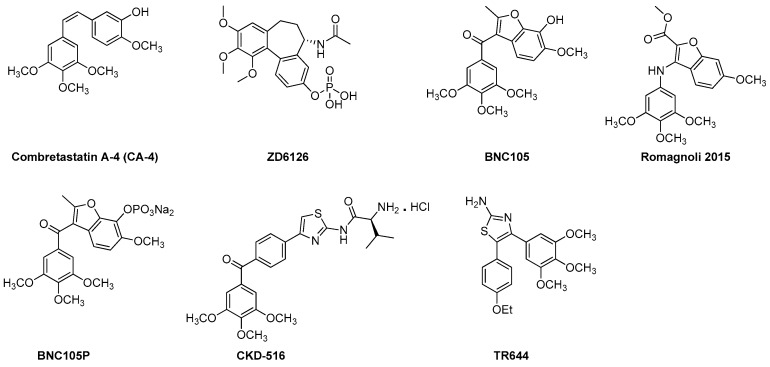
Recently developed VDAs and antiangiogenic agents from the CA-4 family.

Combretastatins are a family of naturally occurring stilbenoid phenols and derivatives (Figure 3). They are a well-studied class of small molecule VDAs and antiangiogenic agents with the ability to exert their microtubule destabilizing effects by binding to the colchicine binding site at the β-subunit of tubulin [99,100]. CA-4 class agents are highly cytotoxic against broad spectrum of human tumor cell lines including the ones that are multi drug resistant (MDR) [19,101]. Promising potential as anti-cancer agents and simple structure of this family have brought them to the attention of medicinal chemists. Thus, in recent years, CA-4 family has undergone extensive structural alterations leading to very promising VDAs and antiangiogenic agents including the ones currently in clinical trials [19,92]. In this section we will focus on recently developed, from years 2010 to 2016, VDAs and antiangiogenic agents belonging to CA-4 class of tubulin depolymerizing agents.

#### 3.1.1. **ZD6126**

Amongst the CA-4 family, the compound that has been extended for further development is the phosphate prodrug **ZD6126** [19,55]. **ZD6126** is known as a water soluble prodrug of the tubulin polymerization inhibitor *N*-acetylcolchinol (NAC). In vivo, **ZD6126**, is rapidly hydrolyzed to its active metabolite, NAC, that occludes tumor blood vessels resulting in shutdown of tumor blood flow and, thus, induces tumor cell death [19,101]. **ZD6126** is highly effective in shutting down newly formed blood vessels right after injection. In experimental tumor models, **ZD6126** has been found to induce rapid blood vessel shut down and significant tumor necrosis at doses of 25–50 mg/kg [19,102,103]. In a phase 1 clinical trial a **ZD6126** dose of up to 80 mg/m^2^ was well tolerated [19]. This compound exhibited higher vascular disrupting effects than antimitotic activity [19]. However, its clinical development has been put on hold due to severe cardiotoxicity [55].

#### 3.1.2. **CKD 516**

**CKD 516** was obtained as a potent tubulin polymerization inhibitor through structural modification of CA-4 [104,105]. It is highly cytotoxic against a number of malignant cell lines including the MDR positive cell lines [19]. It exhibits significant vascular disruption leading to rapid tumor necrosis at a dose of 0.7 mg/kg [19,106]. Thus, it is a highly potent VDA capable of exerting its effect through tubulin component of the tumor vessel. A recent phase 1 clinical trial of **CKD 516** demonstrated that it is safe and well tolerated in the patients with advanced solid tumors at the maximum tolerated dose (MTD) of 12 mg/m^2^, administered on D1 and D8 every three weeks [107].

#### 3.1.3. **BNC 105** and **BNC 105P**

**BNC** causes damage to existing tumor vessels by binding to the colchicine binding site of tubulin [108]. **BNC 105** demonstrates 80-fold more selectivity against proliferating endothelial cells as compared to CA-4 that does not show any selectivity [19]. **BNC 105** has been found to cause 95% vascular disruption at 1/8th of its no observed adverse event level (NOAEL) and, thus, has emerged as a potent disruptor of tumor vasculature [19]. It is currently undergoing a phase I clinical trial.

**BNC 105P** is a disodium phosphate prodrug of **BNC 105** that exhibits the capability of disrupting the vasculature of solid tumors in mice [109]. In vivo, at a dose of 10 mg/kg **BNC 105P** exerts greater vessel disruption (>95%) than **BNC 105** alone [109]. Currently, it is undergoing a phase I clinical trial.

#### 3.1.4. Benzofuran CA-4 Derivative

Recently, Ramognoli et al. reported the discovery of a highly potent benzofuran microtubule destabilizing agent capable of exerting its tubulin depolymerization effect by binding to the colchicine binding site of the tubulin [91]. It demonstrated strong vascular disrupting properties both in vitro and in vivo as well as potent antitumor activity in vivo in a murine model at a dose of 30 mg/kg.

#### 3.1.5. **TR644**

**TR644** is an analogue of CA-4 family with superior microtubule destabilizing effect compared to the lead compound. It exerts it tubulin depolymerization effects by binding to the colchicine binding site of tubulin [92]. It was tested in human umbilical endothelial cells (HUVEC) for antiangiogenic effects and it showed promising inhibitory effect against capillary tube formation as well as endothelial cell migration at a single dose of 30 mg/kg after 24 h of treatment. **TR644** was found to exert its anti-vascular activity through G2/M phase arrest in endothelial cells.

### 3.2. Miscellaneous Recent VDAs and Antiangiogenic Agents

#### 3.2.1. Plinabulin (**NPI-2358**)

**Figure 4:**
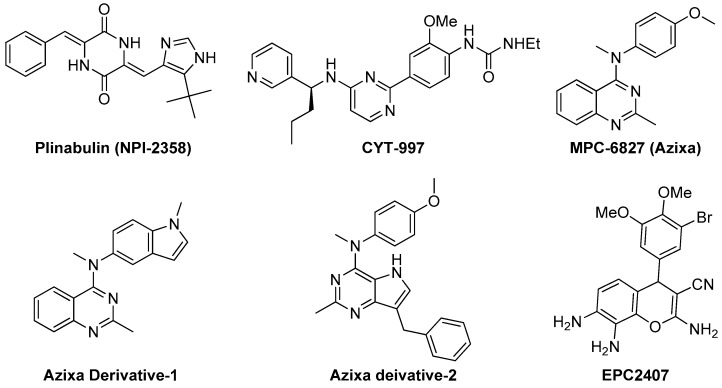
Examples of recently developed non CA-4 VDAs.

Recently, Yakushiji et al. designed and developed a synthetic derivative of the amrine product diketopiperazine, named pinabulin (**NPI-2358**, Figure 4) [110]. **NPI-2358** is a potent microtubule destabilizing agent that exerts its effect by binding to the colchicine binding site of tubulin. **NPI-2358** projects its potent antitumor activity against a broad spectrum of tumor cell lines including DU145, NCI-H292, MDA-MB-231, and PC-3 [19]. **NPI-2358** has also been found to demonstrate potent antitumor activity in animal models treated by it. A phase-I study demonstrated its promising vascular disrupting effects by inducing strong microtubule destabilization, at doses from 13.5 mg/m^2^ to 30 mg/m^2^ in combination with 75 mg/m^2^ docetaxel [111]. It is currently undergoing a phase-II clinical trial with docetaxel.

#### 3.2.2. **CYT-997**

Recently Burns et al. designed and synthesized **CYT-997** (Figure 4), a highly cytotoxic pyrimidine derivative that works against a range of cancer cell lines through microtubule destabilization [112]. It is also equally effective in a broad spectrum of in vivo tumor models. **CYT-997** exhibits strong potential as a VDA inducing rapid and significant morphological changes in human endothelial cells as well as shutdown of blood flow to the tumors at concentrations 65 mg/m^2^ and above [19,113]. It also promotes extensive ablation of the tumor vasculature preventing the tumor from enlarging. It is seen to significantly extend survival in vivo in a murine model with aggressive myelomatosis [114].

#### 3.2.3. Azixa and Its Derivatives

The 4-arylaminoquinazoline Azixa (**MPC-6827** or verbulin, Figure 4), emerged a few years ago as a highly potent tubulin depolymerizing agent capable of inducing cell cycle arrest and cell death achieved via microtubule destabilization [115]. It also found to act as a VDA inducing rapid shutdown of tumor blood flow resulting in inhibition of tumor growth. It works at a low nanomolar range against a variety of tumor cell lines, including brain cancer, melanoma, small cell lung cancer, prostate cancer, and pancreatic cancer [116,117,118]. Recently, it is reported that **MPC-6827** induces rapid tumor vessel occlusion and massive tumor necrosis in the OVCAR-3 xenograft models [19]. It is noteworthy to mention that **MPC-6827** has been found to easily penetrate blood brain barrier and quickly distribute to the central nervous system [14]. This success makes it an outstanding candidate against brain metastasis and CNS malignancies with negligible CNS toxicities. The indole derivative of **MPC-6827**, **Azixa derivative-1** (Figure 4) shows highly potent activity against a number of malignant cell lines with IC_50_ values in the single digit nanomolar range [118]. This compound has been noticed to induce suppression of vessel-like tube formation in endothelial cells as well as destruction of existing blood vessels both in vitro and in vivo at the concentration of 5 mg/kg, resulting in rapid necrosis of tumor cells [19]. Recently, Gangjee et al. reported the design, synthesis and evaluation of another Azixa analogue (**Azixa derivative-2**, Figure 4) exhibiting both cytotoxic and antiangiogenic activity [23]. The compound produces its cytotoxic activity through microtubule destabilization by binding to the colchicine site of tubulin. The same compound exerts antiangiogenic activity in vivo through VEGFR2 inhibition at a dose of 30 mg/kg or above. This compound induced rapid reduction in vascularity and tumor size in two flank xenograft model (the BLBC MDA-MB-435 and U251 glioma models) and in a 4T1 triple negative breast orthotopic allograft model.

#### 3.2.4. **EPC2407**

Recently, EpiCept developed **EPC2407** (Figure 4) a 4*H*-chromene analogue that exerts breast cancer apoptosis and cell cycle arrest in G2/M phase by binding to the colchicine binding site of tubulin [19,119]. **EPC2407** is noticed to disrupt the capillary tube formation in endothelial cell in vitro as well as destroy functional vasculature in vivo at a single dose of 10 mg/kg, inducing rapid necrosis [19]. It is currently undergoing a phase-II clinical trial. Baed on this success, our group also recently reported a number of 4*H*-chromene and chromenopyridine analogues with potential antiangiogenic or VDA activity [24,119,120].

## 4. Conclusions and Future Directions

Recent research in nanoparticle drug delivery of tubulin inhibitors has proven to introduce positive changes in the pharmacokinetics as well as pharmacodynamics of the chemotherapeutics through: (a) improving solubility; (b) enhancing half-life by avoiding first pass kidney clearance; (c) greatly augmenting drug concentrations in tumor cells by improved permeability and retention effects; (d) protecting the drug from enzymatic degradation, as well as (e) circumventing toxicity. Currently, further research has been carried out to overcome the limitations of drug load capacity. In the context of VDAs and antiangiogenic agents, tubulin inhibitors targeting the colchicine binding site have become increasingly popular. Considerable progress has been made in both industrial and academic arenas with more than ten compounds entering in clinical trials as VDAs. One of the biggest advantages of these VDAs over other commonly used chemotherapeutics is their ability to bind reversibly to the colchicine binding pocket and, thus, their reduced toxicity. However, there are still some efficacy and toxicity limitations. Thus, current medicinal chemistry alterations are being carried out focusing on reduction of toxicities and improvement of efficacies related to VDAs.

## Figures and Tables

**Table 1:** Nanoparticle mediated delivery of tubulin inhibitors.

Drugs	Formulation	Active/Passive	Advantage	References
Tubulysine A	Thiol derivative of TubA attached to a linear hexacyclodextrin based polymer via a disulfide linker leading to stable nanoparticles (CDP-TubA)	Active	In vivo CDP-TubA showed a potent antitumor effect and significantly prolonged survival compared with TubA alone	Schluep et al. [29]
Emtansine (DM1)	DM1 loaded PLA-TPGS copolymer nanoparticles immobilized with folates (FA-DM1-NPs)	Active	FA-DM1-NPs induce rapid apoptosis avoiding toxicities, side effects and nonspecific distributions exerted by DM1 alone	Tang et al. [46]
curcumin	α-Cyclodextrin	Active	α-cyclodextrin-curcumin complex selectively enters human lung cancer cell (A549) as compared to the human normal lung fibroblast (WI38) and delivers hydrophobic curcumin	Jana et al. [55]
Colchicine alkaloid	PEGylated Cationic Liposomal-colchicine (PCL-colchicine) nanoparticles	Passive	Microtubules are more efficiently disrupted by nanoparticle-loaded colchicine. In vivo longer survival time for the PCL-colchicine treated group	Tangutoori et al. [56]
**LY293**	Biodegradable co-polymer, mPEG-b-P (CB-co-LA)	Passive	**LY293** loaded nanoparticles demonstrated highly promising efficacy against resistance melanoma cells both in vitro and in vivo without noticeable toxicities to the important organs	Mundra et al. [26,28]
Combretastatin A-4 (CA4)	Multi drug delivery system (DDS) based on mesoporous silica nanoparticles (MSNs) followed by anchoring the MSNs onto the iRGD peptide	Active	1. Co-loading of antiangiogenic CA4 and chemotherapeutic Dox in the MSNs. 2. CA4 is released from the DDS rapidly and target specifically at the tumor vasculature. Later the Dox gets released predominantly within the cells of low pH	Li et al. [64]
Combretastatin A-4 (CA4)	Dendron-polymer conjugates (DPDs)	Passive	The DPDs-CA4 construct has shown far superior cytotoxicity than the DPSs alone in the in vitro cellular internalization and toxicity studies	Sanyal et al. [65]
Etoposide	Solid lipid nanoparticles (SLN)	Passive	Circumvented the issues associated with its low solubility as well as the low bioavailability. in vivo significant reduction in the metastasized tumor colonies as compared to the etoposide alone	Athawale et al. [27]
Paclitaxel	Albumin-bound paclitaxel (nab-pac)	Active	Activity of nab-paclitaxel against pediatric models of rhabdomyosarcoma and neuroblastoma revealed noticeable in vivo activity superior to that of paclitaxel	Houghton et al. [67]
Paclitaxel	polysorbate 80/Tween 80 (P80) coated BSA-paclitaxel	Passive	In vivo experiments exhibited that P80 coated BSA-paclitaxel reached the mouse brain in significantly high concentrations than either the uncoated BSA-paclitaxel or paclitaxel itself	Bansal et al. [82]
Paclitaxel	Pep-1-conjugated PEGylated nanoparticles (Pep-NP-PTX)	Active	Pep-NP-PTX system has been uptaken by the glioma cells at significantly higher amount than the NP-PTX system	Wang et al. [83]
